# Cryo-EM structure of the plant nitrate transporter AtCLCa reveals characteristics of the anion-binding site and the ATP-binding pocket

**DOI:** 10.1016/j.jbc.2022.102833

**Published:** 2022-12-26

**Authors:** Jin He, Mingxing Wang, Shanshan Li, Long Chen, Kaiming Zhang, Ji She

**Affiliations:** 1 MOE Key Laboratory for Cellular Dynamics, School of Life Sciences, Division of Life Sciences and Medicine, University of Science and Technology of China, Hefei, China; 2 Biomedical Sciences and Health Laboratory of Anhui Province, University of Science and Technology of China, Hefei, China

**Keywords:** CLC, nitrate, AtCLCa, cryo-EM, ATP, antiporter, vacuole, AtCLCa, *Arabidopsis thaliana* CLCa, CBS, cystathionine ß-synthase, CLC, chloride channel, CV, column volume, DDM, n-dodecyl-β-d-maltopyranoside, EcCLC, *Escherichia coli* CLC, GDN, glycol-diosgenin, TMD, transmembrane domain

## Abstract

Nitrate is one of the major nitrogen sources for most plants. Chloride channel (CLC) proteins mediate the transport and vacuole storage of nitrate in plants, but the structural basis of nitrate transport by plant CLC proteins remains unknown. Here, we solved the cryo-EM structure of ATP-bound *Arabidopsis thaliana* CLCa (AtCLCa) at 2.8 Å resolution. Structural comparison between nitrate-selective AtCLCa and chloride-selective CLC-7 reveals key differences in the central anion-binding site. We observed that the central nitrate is shifted by ∼1.4 Å from chloride, which is likely caused by a weaker interaction between the anion and Pro160; the side chains of aromatic residues around the central binding site are rearranged to accommodate the larger nitrate. Additionally, we identified the ATP-binding pocket of AtCLCa to be located between the cytosolic cystathionine β-synthase domains and the N-terminus. The N-terminus may mediate the ATP inhibition of AtCLCa by interacting with both ATP and the pore-forming transmembrane helix. Together, our studies provide insights into the nitrate selectivity and ATP regulation of plant CLCs.

Members of the chloride channel (CLC) family play an important role in anion transport in bacteria, animals, and plants. *Escherichia coli* CLC (EcCLC) functions as a chloride/proton antiporter (1). In animals, the CLC family contains both CLCs and chloride/proton antiporters (2). While animal CLC channels localize on the plasma membrane, the antiporters reside in intracellular membranes and catalyze the exchange of two Cl^−^ ions for one proton (3, 4). Animal CLCs have been shown to regulate the stabilization of membrane potential, transepithelial transport, muscle excitability, endolysosomal ion homeostasis, etc. (2) Similar to animal antiporters, plant CLCs reside in various intracellular organelles (5, 6), including vacuoles, mitochondria, Golgi apparatus, and chloroplasts. In *Arabidopsis thaliana*, seven CLC isoforms (AtCLCa-AtCLCg) are known, among which *A. thaliana* CLCa (AtCLCa) is the best characterized (6, 7). AtCLCa is a 2NO_3_^−^/1H^+^ antiporter on vacuoles that drives the uptake of nitrate by a pH gradient across the membrane (8).

Nitrate is one of the major nitrogen sources for most plants (9). AtCLCa mediates nitrate storage in plant vacuoles (8). AtCLCa mutations result in a reduction of approximately 50% in nitrate accumulation in plants (10, 11). The activity of AtCLCa is regulated by nucleotides. ATP inhibits the transport activity of AtCLCa, while AMP prevents this inhibition by competing with ATP (12). Moreover, AtCLCa expression is highest during the dark (13). It was thus suggested that nitrate storage mediated by AtCLCa takes place during the night when ATP:AMP-regulated nitrate assimilation is low. Along with AtCLCa, AtCLCb (13) and AtCLCe (14) were also reported to participate in maintaining plant nitrate homeostasis. Additional functions associated with plant CLCs include cytosolic pH regulation (15), stomatal closure (16), salt stress tolerance (17, 18), and pathogen defense (19, 20, 21).

Structural and functional studies of CLC proteins have provided important insights into their anion selectivity and transport (22, 23, 24, 25, 26). CLC protein forms a homodimer in which each protomer contains its own ion transport pathway, in which three anion-binding sites were identified (23, 25). Gating glutamate (Glu_gate_) controls ion transport by changing its side chain position in the transport pathway. Moreover, the serine at the central binding site (Ser_c_) contributes to the formation of an intracellular kinetic energy barrier for chloride permeation (25). Previous studies have reported that Pro160, which replaces Ser_c_ in animal CLCs, is crucial for the nitrate selectivity of AtCLCa (11, 27). However, the structural basis for the nitrate transport of plant CLCs has remained elusive. Here, we report the cryo-EM structure of ATP-bound AtCLCa, which reveals the molecular features critical for its nitrate selectivity and ATP regulation.

## Results

### Overall structure of AtCLCa

**Figure 1:**
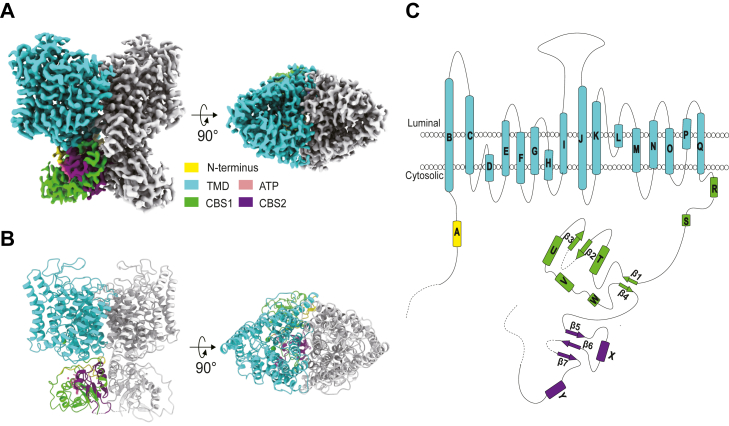
**Overall structure of AtCLCa.***A*, cryo-EM density map of the AtCLCa dimer with one subunit colored *gray* and the other colored by individual structural elements. *B*, cartoon representation of AtCLCa in the same orientation and colors as the density map in A. *C*, domain topology of the AtCLCa subunit. AtCLCa, *Arabidopsis thaliana* CLCa.

Full-length AtCLCa was expressed in a suspension culture of HEK293F cells using the BacMam system. The protein was purified to homogeneity in glycol-diosgenin (GDN) detergent and analyzed by cryo-EM. The structure of AtCLCa was determined to a resolution of 2.8 Å, allowing confident model building of most of the protein regions (Fig. 1, Table S1 and Figs. S1–S3). Similar to the structures of other CLC proteins (22, 23, 24, 25, 26), AtCLCa forms a homodimer displaying C2 symmetry (Fig. 1, *A* and *B*). Each subunit of the dimeric protein is composed of a transmembrane domain (TMD) and a cytoplasmic domain (Fig. 1, *B* and *C*). The cytoplasmic domain consists of the N-terminus and two C-terminal cystathionine ß-synthase domains (CBS1 and CBS2). Both the TMD and the cytoplasmic domain contribute to the dimeric interface of AtCLCa. As in the structures of CLC-7 and CLC-5 (25, 28), the ATP-binding pocket is formed by the cytoplasmic domain (Fig. 1, *A* and *B*). Despite the low sequence identity between AtCLCa and chicken CLC-7 (29.3%) (Fig. S4), the structures of the two proteins can be well aligned with an RMSD of 2.1 Å over 1058 Cα (Fig. S5).

### Ion transport pathway

**Figure 2:**
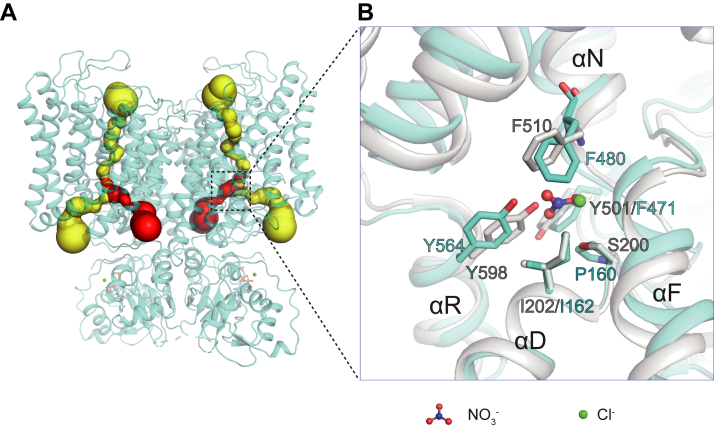
**Ion transport pathways of AtCLCa.***A*, each subunit of AtCLCa contains a NO_3_^−^-conduction pathway (*yellow*) and a H^+^-conduction pathway (*red*). *B*, structural comparison of the central anion-binding site of AtCLCa (*cyan*) and chicken CLC-7 (PDB ID 7JM6, *gray*). AtCLCa, *Arabidopsis thaliana* CLCa; CLC, chloride channel.

The nitrate/proton transport pathway is located within each TMD of the dimeric transporter (Fig. 2*A*). The side chain of Glu_gate_ (Glu203) adopts the ‘up’ conformation, pointing toward the luminal side (Fig. S6). The transport pathway of AtCLCa is divided into an NO_3_^−^-conduction pathway and an H^+^-conduction pathway on the cytosolic side (Fig. 2*A*). Given the high structural similarity of the transport pathway between AtCLCa and EcCLC (Fig. S6), AtCLCa should explore an ion exchange mechanism similar to that of other CLC transporters (24, 29). Three nonprotein densities can be identified in the map with intensities of ∼15 σ, ∼13 σ, and ∼12 σ, corresponding to ions bound at the external, central, and internal binding sites, respectively (Fig. S7). The ions are most likely nitrate because we purified the protein in a buffer containing 150 mM sodium nitrate instead of sodium chloride.

AtCLCa is a 2NO_3_^−^/1H^+^ antiporter with high selectivity for nitrate over chloride (8), and Pro160 in the central binding site has been identified as the key residue for nitrate selectivity (11, 27). For example, in a vacuole patch-clamp study, the nitrate current density for WT AtCLCa was shown to be approximately threefold larger than that for chloride, while the nitrate current density became approximately two-fold smaller than that for chloride when Pro160 was mutated to serine (11). For comparison, ClC-7/Ostm1 currents decreased by ∼30% upon replacing extracellular Cl^−^ with nitrate (30). To understand the molecular basis of the nitrate selectivity, we docked nitrates in the structure of AtCLCa using AutoDock Vina (31, 32). Three nitrate-binding sites were reliably predicted along the transport pathway: they are close to the known chloride-binding sites (23, 24) and overlap well with the EM densities (Fig. S7). The structural comparison between AtCLCa and chicken ClC-7 reveals differences in the central anion-binding site (Fig. 2). In chicken CLC-7 and EcCLC, the central ion-binding site is formed by Ser_c_ (Ser200 in chicken CLC-7), a tyrosine (Tyr598 in chicken CLC-7), and the N-terminal end of helix αN (23, 25). In AtCLCa, nitrate is similarly coordinated by the side chain of Tyr564 and backbone nitrogen atoms from the N-terminal end of helix αN, but a hydrogen bond cannot be formed between the anion and the side chain of Pro160, which likely explains the weakened chloride preference. The nitrate center shows an ∼1.4 Å shift from the chloride ion in CLC-7 (Fig. 2*B*). Additionally, the side chains of Phe471, Phe480, and Tyr564 around the central binding site exhibit noticeable outward shifts relative to the anion (Fig. 2*B*), which seems to be structural rearrangement to accommodate the larger nitrate. These structural changes provide an explanation for the nitrate preference of AtCLCa.

### ATP-binding pocket

**Figure 3:**
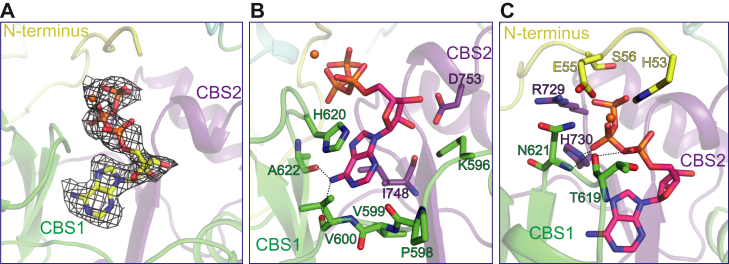
**ATP binding in AtCLCa.***A*, ATP-binding site in the cytoplasmic domain of AtCLCa. The cryo-EM density of ATP is shown as a *brown mesh* (contoured at 15σ). *B* and *C*, two different views of the ATP-binding site in the cytoplasmic domain of AtCLCa. Mg^2+^ is shown as an *orange sphere*. AtCLCa, *Arabidopsis thaliana* CLCa.

Mg^2+^-bound endogenous ATP can be modeled in a large nonprotein density located at the interface of the N-terminus and the two CBS domains (Figs. 1*B*, 3*A* and Fig. S3). Sandwiched between His620 and Ile748 (Fig. 3*B*), the adenine group of ATP binds deeper in the pocket than in the pocket of chicken CLC-7 (Fig. S8) (25). The carbonyl groups of Ala622 and Val600 interact with the amino group of the adenine base, which determines the nucleotide selectivity (Fig. 3*B*). Pro598, Val599, and Val600 in the linker of CBS1 and CBS2 interact extensively with the ring of the adenine base. The hydroxyl groups of the ATP ribose form polar interactions with Asp753 and Lys596 (Fig. 3*B*). Consistent with this observation, a previous study showed that ATP inhibition of AtCLCa was abolished by the mutation Asp753Ala (12). The α-phosphate of ATP forms polar interactions with the carbonyl group of Thr619 of CBS1 and the side chain of His53 (Fig. 3*C*). The β-phosphate forms hydrogen bonds with the main chain nitrogen of Asn621 of CBS1 and the side chain of His730 of CBS2 (Fig. 3*C*). The interactions involving the γ-phosphate will be discussed in the following section. These extensive interactions are consistent with the fact that ATP interacts with AtCLCa with high affinity, explaining their copurification despite extensive washing during purification.

### ATP regulation

**Figure 4:**
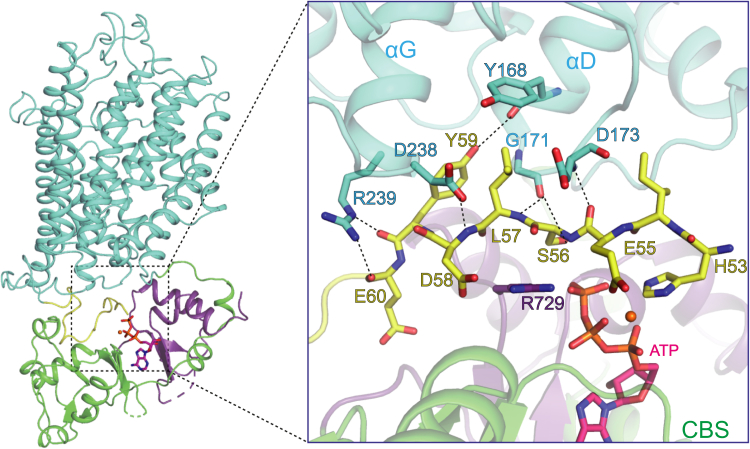
**The N-terminus of AtCLCa interacts with ATP and the TMD.** Mg^2+^-bound ATP is shown in *orange*, and residues in the N-terminus are shown as *yellow sticks*. Polar interactions between the N-terminus (*yellow*) and TMD (*cyan*) are indicated by *dashed lines*. AtCLCa, *Arabidopsis thaliana* CLCa; TMD, transmembrane domain.

Previous studies of AtCLCa have shown that ATP inhibited the transport activity of AtCLCa by 56%, but ADP and AMP showed no obvious inhibitory effect (12, 16). Thus, the γ-phosphate seems to play a critical role in the inhibition of AtCLCa. In our AtCLCa structure, the γ-phosphate and Mg^2+^ establish multiple polar interactions with the N-terminus of AtCLCa (Figs. 3*C* and 4). The γ-phosphate forms hydrogen bonds with the side chains of Ser56 in the N-terminus and Arg729 of CBS2, which further forms a salt bridge with Asp58 in the N-terminus. Mg^2+^ is coordinated by the triphosphate group of ATP and the side chains of His53 and Glu55 in the N-terminus of AtCLCa (Fig. 4). In addition, His53 interacts directly with the α-phosphate and γ-phosphate. Interactions involving His53 were not observed in previous CLC structures.

Stabilized by the ATP molecule, the N-terminus of AtCLCa interacts with the TMD at two major contact sites (Fig. 4). The first contact site is formed between the main chain atoms of Asp58, Tyr59, and Glu60 in the N-terminus and the side chains of Asp238 and Arg239 preceding helix αG. The second site involves the pore-forming helix αD. The side chains of Leu57 and Tyr59 establish interactions with Tyr168 of helix αD. In addition, the main chain oxygen of Glu55 in the N-terminus establishes a hydrogen bond with the main chain nitrogen of Asp173 in the loop following helix αD, and the hydroxyl group of Ser56 and the main chain atom of Leu57 interact with the carbonyl oxygen of Gly171. On the other side of the loop, residues from the C-terminal domain, including Arg724 and Arg751 of the CBS2 domain, further stabilize it through polar interactions (Fig. S9*A*). Notably, helix αD is a pore-forming helix, and key residues of the transport pathway, including Pro160 and Ile162, are located in the loop preceding this helix (Fig. 2). It is therefore plausible that the N-terminus, interacting with both ATP and the TMD, mediates the ATP inhibition of AtCLCa. Coinciding with our observation in AtCLCa, the N-terminus of human CLC-7, sandwiched between the CBS domain and TM domain, has been shown to regulate the transport of CLC-7 (26). The mutations of Asp98 and Tyr99 in CLC-7, corresponding to Asp58 and Tyr59 in AtCLCa, resulted in larger current and fast activation kinetics, while the mutation of Glu95 in CLC-7, corresponding to Glu55 in AtCLCa, increased activation kinetics.

## Discussion

CLC proteins play a critical role in plant nitrate storage (8, 10). Our studies reveal structural differences between nitrate-selective AtCLCa and chloride-selective CLC-7, whereby nitrate shows an obvious shift from chloride at the central site and is accommodated in a binding site surrounded by several aromatic residues. Replacing serine with Pro160 in the transport pathway weakens the binding of chloride and thus reduces chloride preference. Although CLC paralogs from plants, such as *A. thaliana*, may have a serine or other residues replacing Pro160 (6), CLC proteins with a conserved Pro160 are seen in a wide spectrum of plants (Fig. S10), indicating their conserved roles in nitrate transport and storage.

AtCLCa has been previously proven to be inhibited by ATP (12, 16). The current study offers structural insights into the ATP regulation of CLCs. The ATP-binding site is located between the N-terminus and the CBS domains of AtCLCa. The N-terminus may affect the transport activity of AtCLCa by causing structural changes in the pore-forming TMD upon ATP binding. In agreement with our structural analysis, the regulatory role of the N-terminus has been shown in a functional study of AtCLCa, in which the phosphorylation of Thr38 at the N-terminus relieved the ATP inhibition of AtCLCa and increased outward anion fluxes across the vascular membrane (16). Moreover, ATP seems to play a major role in stabilizing the cytoplasmic CBS domains. The ATP-bound CBS domains form a homodimer and establish multiple interactions with the N-terminus and the loop following helix αD (Figs. 1, *A* and *B* and S9), likely contributing to the transport regulation of AtCLCa as well. However, in the absence of an ATP-free AtCLCa structure, the conformational changes induced by ATP binding are still unknown. Overall, our structural studies provide a foundation for further mechanistic understanding of plant CLCs.

## Experimental procedures

### Protein expression and purification

AtCLCa (NCBI accession: NP_198905.1) containing a C-terminal 3C cleavage site followed by a strep tag was cloned into a pEZT-BM vector (33) and heterologously expressed in HEK293F cells (Life Technologies) using the BacMam system (Thermo Fisher Scientific). Baculovirus was generated in Sf9 cells (Life Technologies) following a standard protocol and used to infect HEK293F cells at a ratio of 1:40 (virus:HEK293F, v/v); the medium was supplemented with 10 mM sodium butyrate to boost protein expression. The cells were cultured in suspension at 37 °C for 72 h and collected by centrifugation at 6000 *g*. The cell pellet was resuspended in buffer A (20 mM Tris, pH 8.0, 150 mM NaNO_3_), supplemented with a protease inhibitor cocktail (containing 2 μg/ml DNase, 0.5 μg/ml pepstatin, 2 μg/ml leupeptin, 1 μg/ml aprotinin, and 0.1 mM PMSF) and homogenized by sonication on ice. AtCLCa was extracted with 1% (w/v) n-dodecyl-β-d-maltopyranoside (DDM, Anatrace) supplemented with 0.2% (w/v) cholesteryl hemisuccinate (Sigma Aldrich) by gentle agitation for 2 h. After extraction, the supernatant was collected after a 60-min centrifugation at 30,000*g* and purified with Strep-Tactin Sepharose (IBA). The resin was then washed successively with five column volumes (CVs) of buffer B (20 mM Hepes, pH 7.5, 150 mM NaNO_3_) plus 0.1% DDM, 5 CVs of buffer B plus 0.05% DDM and 0.03% GDN (Anatrace), 5 CVs of buffer B plus 0.02% DDM and 0.048% GDN, and 5 CVs of buffer C (20 mM Hepes, pH 7.5, 150 mM NaNO_3_, and 0.06% GDN). The protein was eluted with buffer C supplemented with 5 mM D-dethiobiotin (Sigma Aldrich) and concentrated to a final volume of ∼500 μl. The sample was further purified by size-exclusion chromatography on a Superdex 200 column (GE Healthcare) preequilibrated with buffer C. The peak fractions were pooled and concentrated to 4.2 mg/ml for cryo-EM analysis.

### EM data acquisition

The cryo-EM grids were prepared by applying AtCLCa (4.2 mg/ml) to a glow-discharged Quantifoil R1.2/1.3 200-mesh copper holey carbon grid (Quantifoil, Micro Tools GmbH). Grids were blotted for 3.5 s under 100% humidity at 4 °C before being plunged into liquid ethane using a Mark IV Vitrobot (Thermo Fisher Scientific). Micrographs were acquired on a Titan Krios microscope (Thermo Fisher Scientific) operated at 300 kV with a K3 Summit direct electron detector (Gatan). Images were recorded with EPU software (Thermo Fisher Scientific) in counting mode with a pixel size of 0.82 Å. The defocus range was set from −1.5 to −3.0 μm. Each micrograph was dose-fractionated to 40 frames under a dose rate of 20 e-/Å^2^/s, with a total exposure time of 3 s.

### Image processing

Two datasets were collected for the AtCLCa sample. All the steps of image processing were performed using cyroSPARC (34). Micrographs were motion corrected with Patch motion correction. The contrast transfer function parameters of the micrographs were estimated using Patch contrast transfer function estimation. For dataset I, 649,938 particles were automatically picked from 1,439 micrographs using 2D class averages of manually picked particles as templates. After two rounds of 2D classification, 202,946 particles were selected for ab-initio reconstruction with four classes. One good class from ab-initio reconstruction of dataset I (127,892 particles) was selected for nonuniform refinement with C2 symmetry imposed. The resulting 3D reconstruction showed a clear two-fold symmetry with a resolution of 3.4 Å. We then performed one round of heterogeneous refinement with three reference maps generated by low-pass filtering the reconstruction from nonuniform refinement at 15 Å, 20 Å, and 40 Å. One good class with 84,904 particles was selected for further processing. For dataset II, 2,263,735 particles were picked and extracted from 5885 micrographs. After two rounds of 2D classification, 666,716 particles were selected for three parallel runs of ab-initio reconstruction with four, five, and six classes. Particles from good classes of the ab-initio reconstructions were combined and duplicate particles were removed, resulting in a dataset of 449,632 particles. A nonuniform refinement was then performed to obtain an EM map of 3.6 Å. After one round of heterogeneous refinement, one class with 252,425 particles was selected. The particles selected after heterogenous refinement from datasets I and II were then merged for nonuniform refinement, resulting in a 2.9 Å map. One more round of heterogenous refinement was performed, and a good class with 202,231 particles was selected for a final nonuniform refinement to obtain a map with an overall resolution of 2.8 Å.

All resolutions were estimated by applying a soft mask around the protein density and the gold-standard Fourier shell correlation = 0.143 criterion. The local resolution map was calculated by cyroSPARC.

### Model building, refinement, and validation

Model buildings were conducted in Coot (35), and the ggCLC7 structure (PDB accession number 7JM6) was used as a reference. Nitrates were docked in the structure of AtCLCa by using AutoDock Vina (31, 32). Real space model refinement (36) and validation (37) were performed in Phenix. The final structure models include residues 53-626, 636-737, and 744-766. Residues 1-52, 627-635, 738-743, and 767-775 are disordered and not modeled. The statistics of the model geometries were generated using MolProbity (38). MOLE was used to calculate the ion transport pathways of AtCLCa (39). Figures were prepared using PyMol (40) and ChimeraX (41) software.

## Data availability

The cryo-EM map of AtCLCa in this study with its associated atomic model has been deposited in the wwPDB OneDep System under EMD accession code EMD-33088 and PDB ID code 7XA9.

## Supporting information

This article contains supporting information.

## Conflict of interest

The authors declare that they have no conflicts of interest with the contents of this article.
