# Supplementary material for: Cryo-EM structure of the plant nitrate transporter AtCLCa reveals characteristics of the anion-binding site and the ATP-binding pocket
Source: J Biol Chem. 2022 Dec 26;299(2):102833. doi: 10.1016/j.jbc.2022.102833 (PMC9898749; doi:10.1016/j.jbc.2022.102833)
Supplement: Supplemental Table S1 and Figures S1–S10 [file mmc1.pdf]

**Supporting information**

**Supporting Table S1**

**Cryo-EM data acquisition, reconstruction and model refinement statistics.**

| | AtCLCa | |
|---|---|---|
| **Data collection and processing** | | |
| Microscope/Detector | K3 | K3 |
| Image software and collection | EPU | EPU |
| Magnification | | |
| Voltage (kV) | 300 | 300 |
| Electron exposure (e-/Å$^2$) | 65 | 65 |
| Defocus range (μm) | -1.5 to -3.0 | -1.5 to -3.0 |
| Pixel size (Å) | 0.82 | 0.82 |
| Micrographs | 1652 | 5885 |
| **Reconsruction** | | |
| Initial particle images | 649,938 | 2,263,735 |
| Final particle images | 202,946 | 666,716 |
| Symmetry imposed | *C*2 | *C*2 |
| **Refinement** | | |
| Model resolution (Å) | | 2.84 |
| FSC | | 0.143 |
| Model composition | | |
| Non-Hydrogen atoms | | 11028 |
| Protein residues | | 1404 |
| B factors (Å$^2$) | | |
| Protein | | 28.33/65.58/40.42 |
| Ligand | | 29.86/98.83/80.25 |
| R.m.s. deviations | | |
| Bond lengths (Å) | | 0.011 |
| Bond angles (°) | | 1.29 |
| Ramachandran plot | | |
| Favored (%) | | 96.55 |
| Allowed (%) | | 3.45 |
| Rotamer Outlines (%) | | 0.17 |
| Validation | | |
| MolProbity score | | 1.53 |
| Clashscore | | 5.59 |

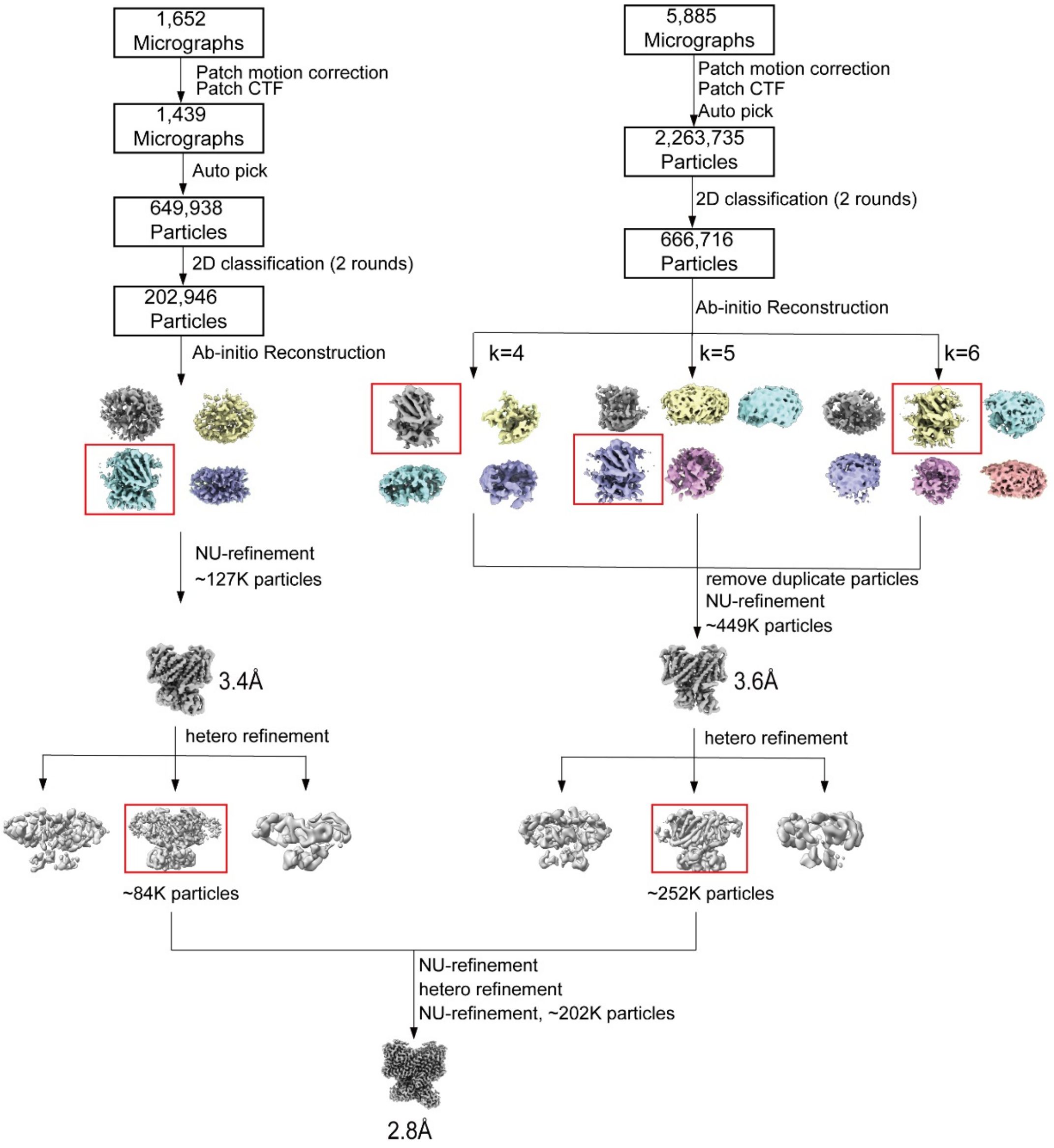


**Supporting Fig. S1**: Flowchart of EM data processing for AtCLCa.

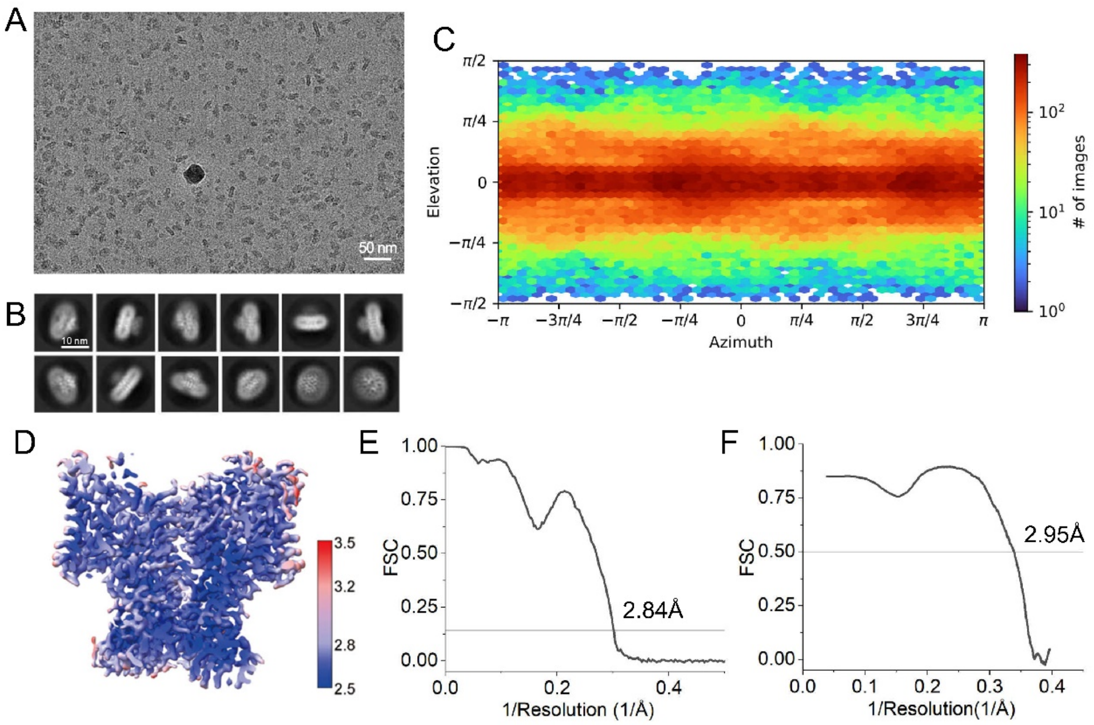


**Supporting Fig. S2**: (A) Representative electron micrograph of AtCLCa. (B) 2D class averages. (C) Euler angle distribution of particles used in the final 3D reconstruction. (D) Final density maps colored by local resolution; (E) Gold-standard FSC curves of the final 3D reconstructions; (F) FSC curves for cross-validation between the model and the map.

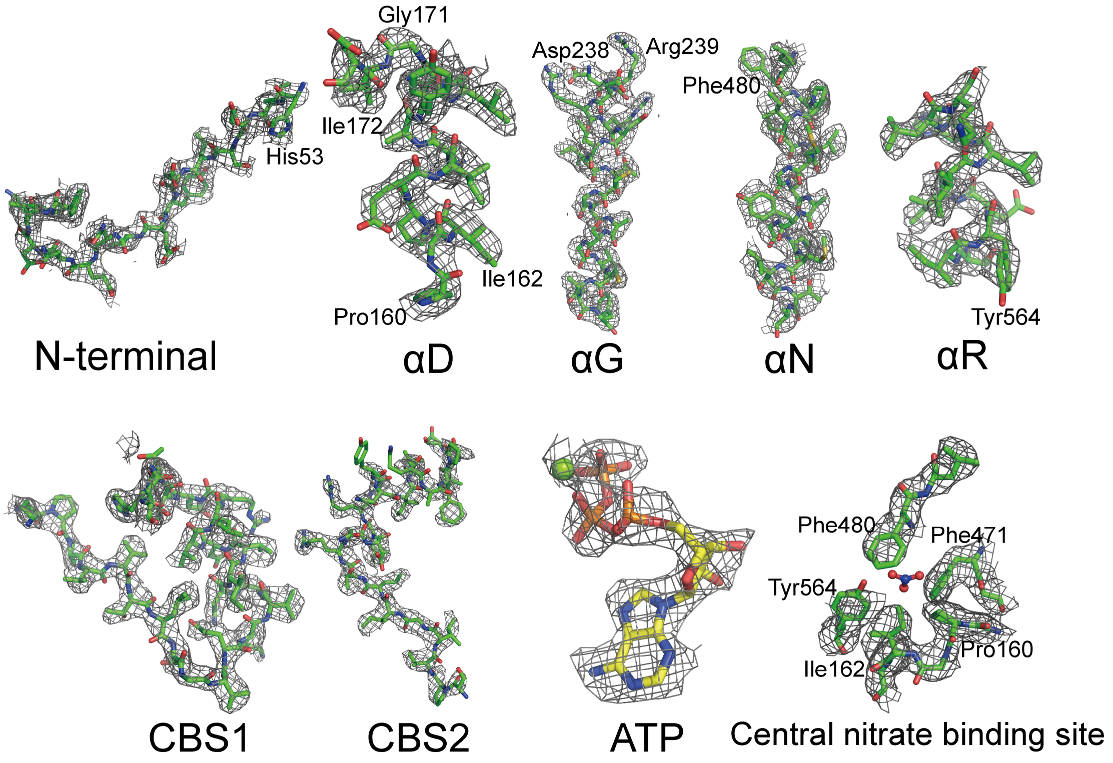


**Supporting Fig. S3:** Representative sections of cryo-EM density shown as grey mesh. Refined coordinates are shown as sticks.

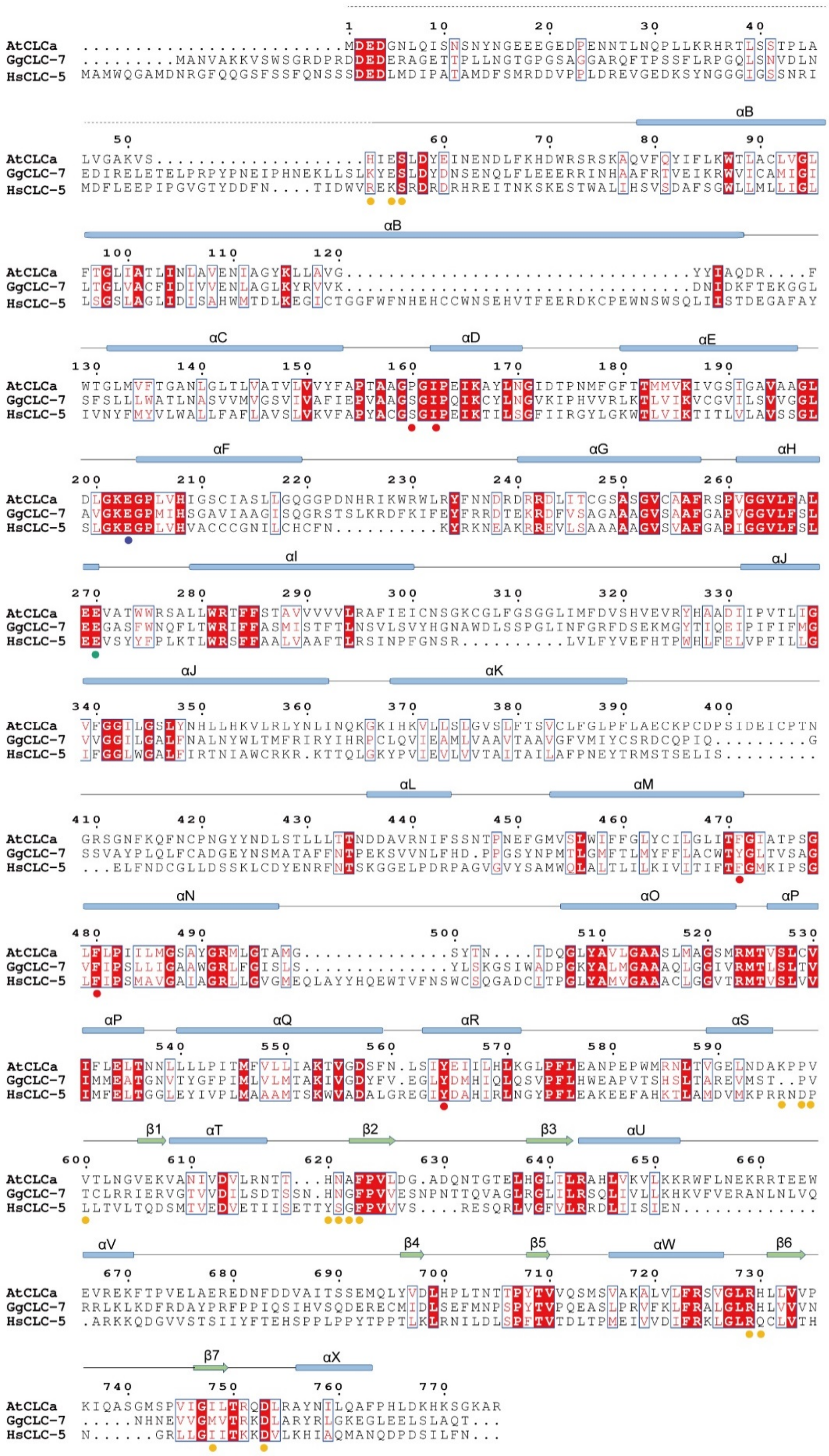

**Supporting Fig. S4:** Sequence alignment of AtCLCa, GgCLC-7, and HsCLC-5. Secondary structure assignments are based on the AtCLCa structure. Orange dots indicate ATP-binding residues; red dots indicate central nitrate-binding residues; a blue dot indicates the $Glu_{gate}$; a green dot indicates the proton glutamate.

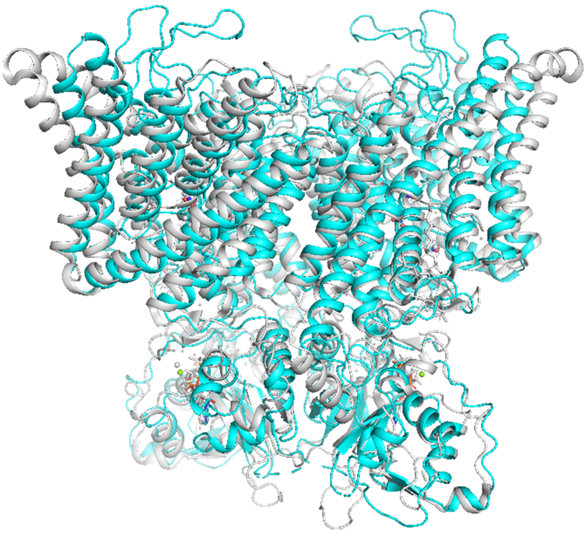

**Supporting Fig. S5:** Structural comparison between AtCLCa (cyan) and chicken CLC-7 (7jm6, grey).

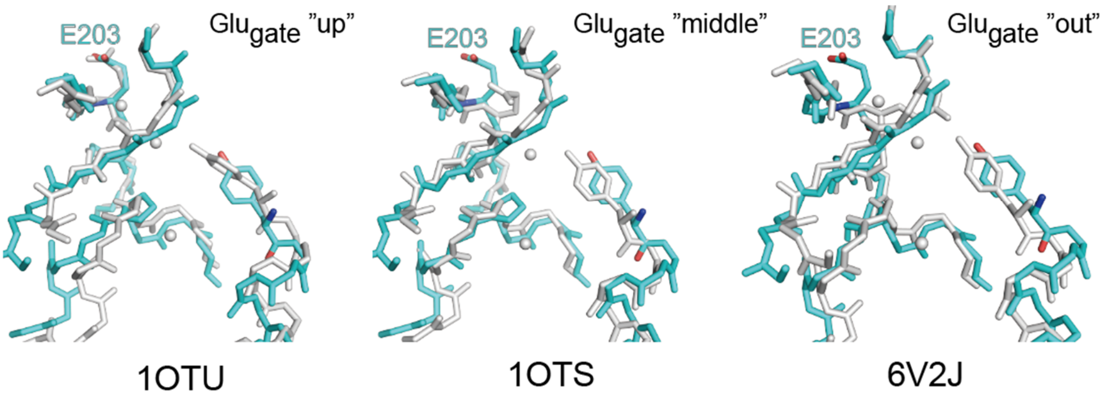


**Supporting Fig. S6**: Comparison of the anion conduction pathway of AtCLCa (cyan) with that of EcCLC (1OTS), EcCLC E148Q (1OTU), and EcCLC QQQ (6V2J). EcCLC, EcCLC E148Q, EcCLC QQQ, and their chloride anions are colored grey.

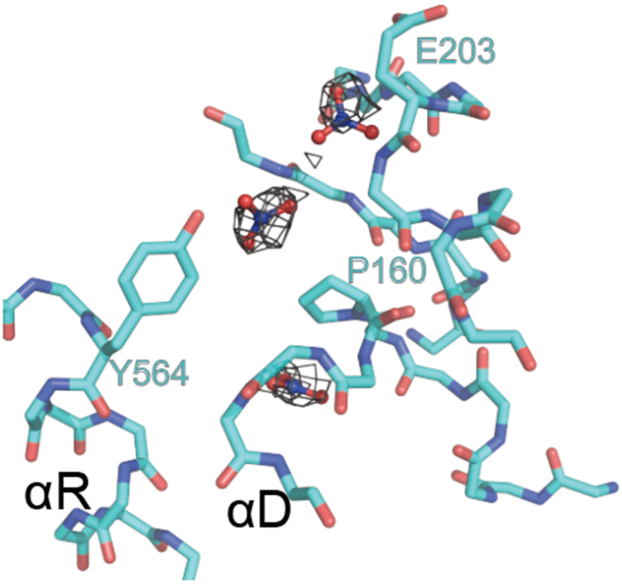


**Supporting Fig. *S7***: Anion binding sites along the AtCLCa ion conduction pathway. EM densities are shown as brown mesh contoured at 8 σ.

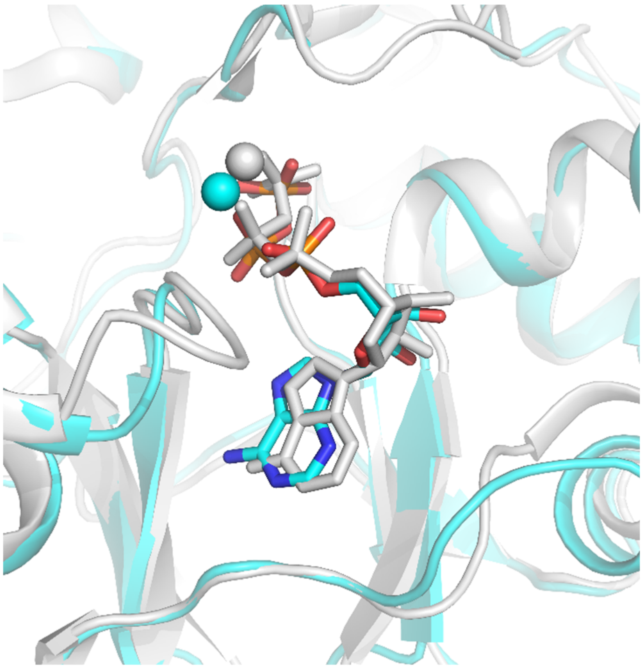

**Supporting Fig. S8:** Superposition of the ATP binding sites of AtCLCa (cyan) and chicken CLC-7 (grey).

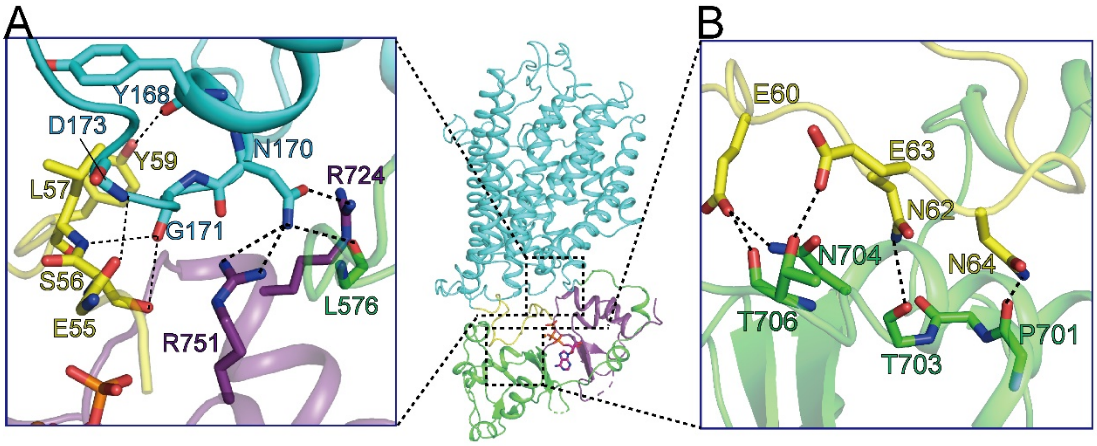


**Supporting Fig. S9:** ATP-bound CBS domains maintain the structural integrity of AtCLCa. (A) Interactions between the CBS2 domain (purple) and TMD (cyan). (B) Interactions between the CBS1 domain (green) and N-terminus (yellow).

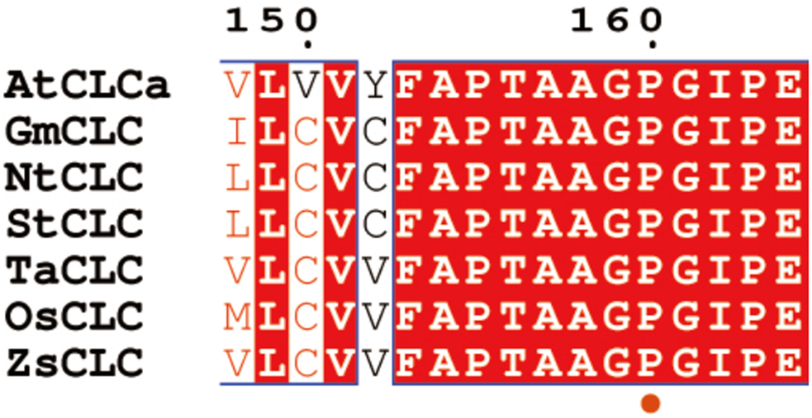


**Supporting Fig. S10:** Sequence alignment of CLC proteins from different plants. Gm, Nt, St, Ta, Os, and Zm represent *Glycine max* (NP_001236494.2), *Nicotiana tabacum* (NP_001312163.1), *Solanum tuberosum* (XP_006357190.1), *Triticum aestivum* (XP_044407782.1), *Oryza sativa* (XP_015620662.1), and *Zea mays* (NP_001385762.1), respectively.
